# Low-salinity water flooding by a novel hybrid of nano γ-Al_2_O_3_/SiO_2_ modified with a green surfactant for enhanced oil recovery

**DOI:** 10.1038/s41598-024-64171-9

**Published:** 2024-06-18

**Authors:** Azin Khajeh Kulaki, Seyed Mojtaba Hosseini-Nasab, Faramarz Hormozi

**Affiliations:** 1 Faculty of Petroleum Engineering, Semnan University, Semnan, Iran; 2 School of Chemical, Petroleum and Gas Engineering, Iran University of Science & Technology, Tehran, Iran; 3 Faculty of Chemical & Petroleum and Gas Engineering, Semnan University, Semnan, Iran

**Keywords:** Hybrid-enhanced oil recovery, Low-salinity water flooding, Nanoparticles, Gum Arabic, Micromodel, Engineering, Chemical engineering, Crude oil

## Abstract

This paper introduces a hybrid enhanced oil recovery (HEOR) method that combines a low-salinity water flooding (LSWF) and nanoparticles (NPs) stabilized with a green surfactant. We experimentally investigated the use of combinations of silica (SiO_2_) and gamma alumina (γ-Al_2_O_3_) nanohybrids stabilized with Gum Arabic (GA) at different water salinities. Nanofluids (NFs) were prepared by dispersing γ-Al_2_O_3_ and SiO_2_ NPs (0.1 wt%) in deionized water (DW), synthetic seawater (SSW), 2, 5, and 10 times diluted samples of synthetic seawater (in short 2-DSSW, 5-DSSW and 10-DSSW, respectively). The challenge is that NPs become unstable in the presence of cations in saline water. Moreover, an attempt was made to introduce NFs with high stability for a long period of time as the optimal NFs. The effects of temperature on the behaviour of optimal NFs in the presence of different base fluids, distinct mass ratios of γ-Al_2_O_3_/SiO_2_ and various concentrations of surfactant were analysed via interfacial tension (IFT) and viscosity measurements. The results of the viscosity measurement showed that with increasing temperature, the NPs dispersed in DW had lower viscosity than NPs dispersed in various salinities. However, the IFT measurement for NPs dispersed in different base-fluids revealed that with increasing temperature and presence of cations in saline water, IFT values decreases. Although, the minimum IFT for hybrid nanofluid (HNF) γ-Al_2_O_3_/SiO_2_ modified with GA and dispersed in 10-DSSW was reported 0.99 mN/m. Finally, according to the micromodel flooding results, in oil-wet conditions, the highest oil recovery for combination γ-Al_2_O_3_/SiO_2_ modified with GA and dispersed in 2-DSSW was reported 60.34%. It was concluded that NFs modified with GA could enhanced applicability of LSWF via delay in breakthrough time and improving sweep efficiency.

## Introduction

According to the forecast made until 2040, use of renewable fuels has been noticed worldwide and fossil fuels such as oil, gas and coal are considered essential energy sources worldwide^1^. In the last few decades, human society has faced impressive growth in population. Therefore, the need for more energy sources is understandable. Limited oil and gas resources have given more attention to enhanced oil recovery (EOR). The LSWF and other EOR methods can be considered new hybrid approaches for improving oil recovery^2–4^. In recent years, to promote oil recovery mechanisms, combining two or more EOR methods, known as hybrid methods, has been explored to address operational challenges, reduce environmental damage and reduce production costs^5^. The HEOR method could be optimized for different injection scenarios and achieve the highest recovery oil factor^6–10^.

NPs play an essential role in the EOR process through four main mechanisms: disjoining pressure, wettability alteration, IFT reduction and viscosity control^4,11–13^. NPs could change the wettability toward more water, combine with LSWF and improve oil recovery^14,15^. The tendency of NPs to aggregate leads to a decrease in their surface energy, which results in the loss of their properties. Therefore, ensuring the stability of NPs in base fluid is the most critical challenge for their application^16^. It is important to evaluate NFs stability before injecting into complex reservoirs. Therefore, NFs stability improvement in reservoir conditions (high temperature, high salinity and etc.) should be carefully considered. If unstable NFs are injected into the reservoir, NPs tend to aggregate resulting they create deposits that could damage to reservoir permeability, throats structures and pores^13^.

Surface modification is performed to minimize the surface energy and reduce the tendency of NPs to aggregate. Physical surface modification could be performed with surfactants or adsorbed macromolecules. In this method, surfactant adsorption occurs on the surface of NPs via electrostatic interactions between polar groups. Surface modification is performed through the modification of covalent bonds on the surface of NPs^17,18^. Adding surfactants to NFs is a cost effective method that may lead to the stability of NFs^19,20^. The completely different structures of surfactants, which are hydrophilic and hydrophobic, allow them to act as bridges between NPs and base fluids^20^.

Torsaeter and Hendraningratt^21^ used Al_2_O_3_, SiO_2_ and TiO_2_ NPs dispersed in 3 wt% brine to displace residual oil in sandstone core samples. According to their results, the combination of SiO_2_ dispersed in brine had the highest duration of stability. Moreover, a maximum oil recovery of 67% was achieved by TiO_2_ NPs with 1 wt% polyvinylpyrrolidone (PVP). Nazari Moghadam et al.^22^ used different NPs to investigate the wettability change in carbonate reservoirs. The imbibition test results revealed the important role of SiO_2_ and CaCO_3_ NPs. The presence of CaCO_3_ and SiO_2_ increases the oil recovery factor by 4 and 6% respectively, of the original oil-in-place (OOIP) ratio. Alomair et al.^23^ used metal oxide NPs including NiO, TiO_2_, Al_2_O_3_ and SiO_2_ at different concentrations. They observed decrease at viscosity in heavy oil when Al_2_O_3_ and SiO_2_ NPs were used at a concentration of 0.05 wt%. Their results showed that the combination of Al_2_O_3_ and SiO_2_ at 0.05 wt% had the highest oil recovery factor among the other NFs. In another work, Alameri et al.^24^ investigated the effect of low-salinity water and surfactants on carbonated core samples. They found that adding surfactant to saline water could improve the oil recovery factor. Al-Anssari et al.^25^ utilized different concentrations of SiO_2_ dispersed in saline water with concentrations of 3, 8, 10, 15 and 20 wt% to investigate wettability changes. Their results revealed the greatest wettable alterations during the first hour of contact between the NFs and the rock surface. Additionally, they reported an optimal salinity range between 3 and 8 wt%. In a similar study, Kiani et al.^26^ used γ-Al_2_O_3_ NPs at different concentrations dispersed at different salinities. Their results showed that the oil recovery factor increases with decreasing salinity. Hou et al.^11^ dispersed SiO_2_ NPs in saline water containing sodium chloride at concentrations of 0.05 and 0.1 wt% to alter the wettability of carbonate rock surfaces. They observed that Na^+^ ions could reach the oil palmitic acid molecules by approaching the carbonate rock surface. In fact, Na^+^ ions allow NPs to replace palmitic acid. thus, wettability alterations occur on the carbonate rock surface. Dehaghani and Daneshfar^14^ applied SiO_2_ NPs and saline water in their studies. They reported the greatest oil recovery factor related to SiO_2_ NPs with 0.25 wt% dispersed in 20 times diluted brine, and the lowest oil recovery factor related to formation water. Wang et al.^27^ reported that the oil recovery factor could be improved to 9% in low-salinity water flooding compared to that in formation water flooding. Their results indicated that the wettability of rock could increase water wetting by using divalent ions such as Mg^2+^, Ca^2+^ and SO_4_^2−^. Additionally, they obtained optimal salinity between 1400 and 3200 mg/L. Moreover, they showed that the relative permeability diagram moved to the right with respect to the initial water saturation during the flooding of low-salinity water and the remaining oil saturation decreased. Bahari et al.^28^ investigated the effects of different surfactants, cetyl trimethyl ammonium bromide (CTAB), sodium dodecyl sulfate (SDS) and PVP on the stability and electrical conductivity of HNFs Al_2_O_3_-SiO_2_. They found that the HNF with a mass fraction of 30:70 has the highest electrical conductivity. Their results showed that the greatest duration of stability was obtained for HNFs prepared using SDS as a surfactant. Mofard and Saeedi Dehaghani^29^ studied the effect of smart water, CTAB and SDS by performing a contact angle test. They found that the combination of surfactant and smart water had the highest displacement efficiency compared to that of individual surfactant flooding or smart water flooding. Zallaghi and Khazali^10^ investigated the effects of CTAB and tridecyl alcohol 30 ethoxylate (TDA 30 EO) surfactants combined with smart water in fractured carbonate reservoirs. They found that oil recovery improved by more than 51% in core floods with strong synergy between smart water and surfactants. Liang et al.^30^ investigated dynamic stability, IFT and oil recovery using the amphiphilic molybdenum disulfide (MoS_2_) nanosheets modified with octadecyl amine (ODA) molecules. The results showed improved stability and reduced IFT to 0.1 (mN/m), where core flooding experiments gave the highest oil recovery of 19.1%.

Table 1 presents some mechanisms and improvements in oil recovery using hybrid methods due to synergistic effects.

**Table 1:** Review of some research performed using the HEOR methods.

Material type	Subject of study	Research achievement	Reference
SiO_2-_MWCNTs HNF	Dynamic viscosity	Dynamic viscosity decrease with increasing temperature	^31^
Al_2_O_3-_MWCNTs HNF	Viscosity measurements	Viscosity of the HNF enhanced with increasing nanoadditives concentration and decreasing temperature	^32^
Al_2_O_3_ and Gum Arabic	Oil recovery	Improving the oil recovery factor by 53%	^33^
Al_2_O_3_ , SiO_2_ Single and HNF dispersed in DW	Termal conductivity and dynamic viscosity	HNF prepared larger values of the viscosity and thermal conductivity in comparison with the single ones	^34^
SiO_2_ and low-salinity water	IFT and Oil recovery	Reducing the IFT to twice-diluted salinity water and improving the oil recovery factor by 6%	^4^
Use of silica gel, Al_2_O_3,_ and iron NPs with salinity water and surfactant	IFT	Reduction of IFT (mN/m) 0.0001	^35^
Low salinity water and Al_2_O_3_ and SiO_2_ NPs	Oil recovery	Improving the oil recovery factor by 4%	^3^
SiO_2_ and SDS surfactant	IFT and oil recovery	The lowest IFT was 0.898 (mN⁄m) and improvement of oil recovery factor to 44.97%	^17^
SiO_2_ and low-salinity water	IFT and Contact angle	The lowest IFT was 14.29 (dyne/cm) and the highest wettability alteration was 126.2*°*	^36^
Smart water and nanocomposite of Al_2_O_3_ and SiO_2_	Oil recovery	Improving the oil recovery factor by 9% to 10%	^6^
Modified seawater and CTAB, SDS and TX-100 surfactants	Contact angle and oil recovery	Reducing the contact angle from 161° to 52° and improving the oil recovery factor to 72%	^7^
Al_2_O_3_ and SiO_2_ HNF with CTAB surfactant	Contact angle and oil recovery	Reducing the contact angle to 19° and improving the oil recovery factor to 92%	^37^
Smart water and surfactant CTAB/TDA 30-EO	Contact angle and oil recovery	Reducing the contact angle to 60° and improving the oil recovery factor to 43.1%	^10^
Smart water and CTAB surfactant	Contact angle	Reducing the contact angle to 43°	^38^
Nanostabilized foam	IFT and oil recovery	reduce the IFT between the oil and water phase and increase the ultimate oil recovery in quasi two-dimensional (2D) porous media	^39^
Nanocomposites silica and alumina based on polyacrylamide (NCSAP), surfactant (CTAB) and polyacrylamide (PAM)	IFT, contact angle and oil recovery	The most effective mechanism in oil recovery for light oil and heavy oil were reported IFT reduction and mobility control, respectively	^40^

In recent years, investigation of HNFs and surfactants has been received much attention by different researchers. However, the stability of NFs in reservoir conditions such as high temperature, high salinity and etc., requires more research and a deeper understanding. In addition to sustainability, using friendly environmental materials in HEOR should be one of the goals to play a key role. The development of nanocomposites modified with green surfactants and dispersed in water with salinity similar to seawater and formation water is greatly essential to improve oil recovery, which should be investigated in future studies. This study aims to investigate the potential effect of a novel γ-Al_2_O_3_/SiO_2_ nanocomposite modified with gum arabic surfactant and dispersed in various salinities and viscosities.

## Experimental materials and equipment

### Experimental materials

SiO_2_ NPs 99.5% pure and 15–20 nm in diameter and γ-Al_2_O_3_ NPs 99% pure and 20 nm in diameter were purchased from US Research Nano-Materials. The mass fraction ratios of γ-Al_2_O_3_ to SiO_2_ NPs were set at 10:90, 30:70 and 50:50. A concentration of 0.1 wt% was used to prepare all the solutions. This number is considered suitable for improving the stability of HNFs and increasing the thermal conductivity^28^. Crude oil with a density of 54.18 lb/ft^3^ (31.47° API) and a viscosity of 25.93 CST was used from the Gachsaran oilfield in Iran for the IFT and flooding experiments (Table 2).

**Table 2:** The composition of crude oil.

Component	Mole %
CO_2_	1.005
H_2_S	0.406
C_1_	36.29
C_2_	6.523
C_3_	5.255
iC_4_	1.07
nC_4_	3.12
iC_5_	1.401
nC_5_	1.805
C_6_	2.896
${C}_{7}^{+}$	40.21

Brines were prepared by dissolving different salts (NaCl, NaHCO_3_, Na_2_SO_4_, and CaCl_2_⋅2H_2_O and MgCl_2_⋅6H_2_O). All the reagents were purchased from Merck (Germany). The brine concentrations were prepared by dissolving salts in water with total dissolved solids (TDS) near 4071, 8142, 20,400 and 40,710 ppm.

Table 3 lists the properties of the brine compositions used in this paper. Hexamethyldisilane, Si_2_(CH_3_)_6_ was utilized to change the wettability of the micromodel from water-wet to oil-wet. Toluene (C_7_H_8_) and methanol (CH_3_OH) were used as wettability-changing agents and washing, respectively, both of which afforded 99% purity.

**Table 3:** Composition of various synthetic water.

Salts (ppm)/brine waters	Seawater (SSW)	Seawater diluted twice (2-DSSW)	Seawater diluted five times (5-DSSW)	Seawater diluted ten times (10-DSSW)	Formation water (FW)
NaCl	28,400	14,200	5680	2840	70,000
MgCl_2_⋅6H_2_O	6340	3215	1268	634	1425
CaCl_2_⋅2H_2_O	1380	690	276	138	20,100
Na_2_SO_4_	4490	2245	898	449	1290
NaHCO_3_	100	50	20	10	1000
TDS (ppm)	40,710	20,400	8142	4071	93,815

GA was used as a surfactant to aid in dispersing the NPs and improving the stability of the suspensions; It was purchased from Merck. GA is a natural polysaccharide obtained from the tree Acacia Senegalese. GA is a multicomponent substance, the majority of which consists of approximately 98 wt% polysaccharide and the smallest fraction consists of 2 wt% protein-polysaccharide. Peptides form the hydrophobic part and polysaccharides form the hydrophilic part of GA. Peptides are adsorbed on the oil surface. However, polysaccharides as the most hydrophilic component, stabilize oil droplets with a negative charge on carboxylates^41,42^. The results of the DLS test conducted by Williams et al.^43^ showed that magnetic NPs modified with GA accumulated less than unmodified NPs after 30 h.

### Experimental equipment

**Figure 1:**
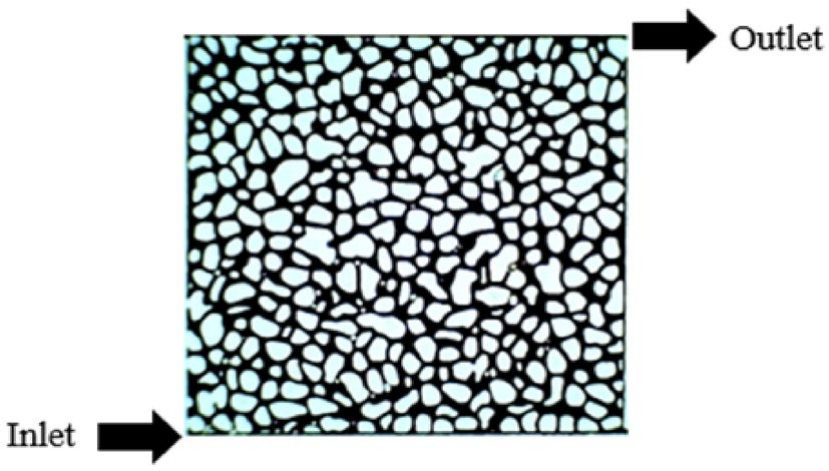
Schematic of the 2D glass micromodel based on the image obtained from the core CT scan^42^. The pore bodies are black and the rocks are white.

A 2D glass micromodel was used as the porous medium and images were obtained from a CT scan of the core^44^. The micromodel was a 2D glass sheet with narrow pores etched on its surface. A piece of engraved glass was placed on the other part of the glass. Then, both parts of the glass were placed in the furnace and fused at 730 °C. The glass sheets bond at high temperatures to form a single piece. Thus, only the engraved pattern and drilled holes are open for flow^14^. The inlet and outlet channels of the micromodel for fluid injection and production are shown in Fig. 1. Additionally, one of the advantages of the glass micromodel is its chemical inertness and its substrate is easily visible^4^. It consists of a 5 × 5 cm^2^ matrix and an etch depth of 460 µm corresponding to a porous media with 41.13% porosity and a 0.51 cm^3^ pore volume (PV).

**Figure 2:**
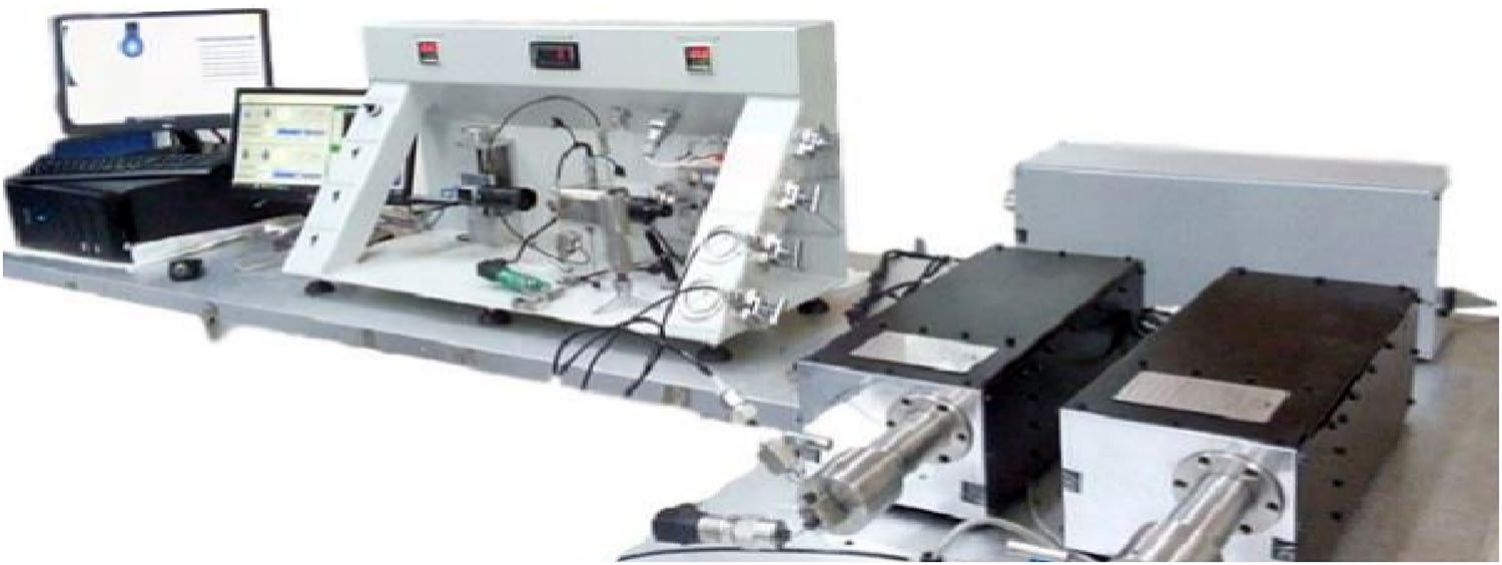
The photo of the IFT measurement device was used for optimal NFs.

Measured IFT between crude oil and NFs dispersed in aqueous phases (DW, SSW, 2-DSSW, 5-DSSW and 10-DSSW) at 25 °C and 60 °C. The system was equipped with a CCD camera and a computerized macro lens. The formed droplets were analysed by professional drop shape analysis software (Apex DSA) based on pendant drop methods. A photo of the IFT device is shown in Fig. 2. After each test, the IFT measurement chambers for crude oil and NFs were washed with distilled water and toluene.

A Brookfield DV3T digital viscometer was used at different temperatures to measure the viscosity of the NFs. The temperature was continuously measured and controlled by a sensor. The spindle ULA was utilized for measuring low viscosity. After selecting the optimal NFs, 16 ml of the 0.1 wt% solution was added to the container with a thermal jacket connected to the rheometer at a constant shear rate of 73 (1⁄S). Before the viscosity of the NFs was measured, distilled water was used to calibrate the device and reduce error.

## Methodology

### Designing experiments

The Taguchi method was used to determine the stability of the nanosolutions in response to low-salinity water (ppm), the mass fraction of γ-Al_2_O_3_/SiO_2_ (wt%) and the surfactant concentration (ppm) as operating variables. The Taguchi method is based on three factors and five levels and was used to determine the optimal process characteristics that minimize the sensitivity^45^. In this research, the first factor was the salinity of the water, the second was the mass fraction of γ-Al_2_O_3_/SiO_2_ and the third was the concentration of GA. According to the factors and their levels in Table 4, an L-25 orthogonal arrangement was used to design the experiments. Since most of the stability was used in the response, a larger qualitative characteristic was chosen as the better criterion. The average values of the two generated responses were considered the original response.

**Table 4:** Factors and levels used to design the experiments with the Taguchi method.

Factors	Level 1	Level 2	Level 3	Level 4	Level 5
Low salinity water (ppm)	1	4071	8142	20,400	40,710
The mass fraction γ-Al_2_ O_3_/SiO_2_ (wt%)	SiO_2_	AS10:90	AS30:70	AS50:50	γ-Al_2_O_3_
GA concentration (ppm)	0	250	500	750	1000

The stability test results were analysed with a signal-to-noise diagram. In this way, if the quality characteristic is large or better, the signal-to-noise ratio (*S/N*) is calculated as follows: The signal-to-noise ratio is calculated from the mean square of the standard deviation (*MSD*), where *Y*_*i*_ is the result of *N* repeated experiments^46,47^.

1 $$ MSD = \left( {\frac{{\frac{1}{{\mathop Y\nolimits_{1}^{2} }} + \frac{1}{{\mathop Y\nolimits_{2}^{2} }} + \frac{1}{{\mathop Y\nolimits_{3}^{2} }} + \cdots + \frac{1}{{\mathop Y\nolimits_{N}^{2} }}}}{N}} \right) $$

2 $$ \left( \frac{S}{N} \right) = - 10Log(MSD) $$

### Preparation of NFs and selection of optimal NFs (screening)

The preparation of hybrid nanofluids (HNFs) involves different steps. Each step has different results. There has been no consensus among researchers about predicting HNFs^18^. In the first step, the solutions were prepared by adding NPs and GA to base fluids with various salinities. Next, the solutions were homogenized by a magnetic stirrer at 500 rpm for 15 min. Finally, to ensure the complete dispersion of the NPs in the base fluids, the mixture was sonicated for 1 h. Due to the instability of NFs in saline water and rapid aggregation, screening was performed. On an operational scale, the aggregation of NPs causes leakage around the injection well, which ultimately causes damage to the formation in the areas around the injection well^48^.

### Micromodel test

**Figure 3:**
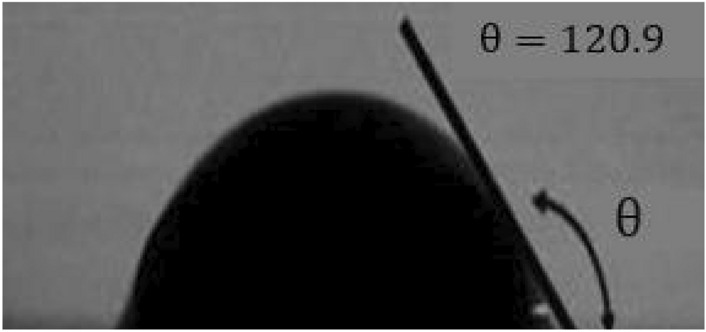
Water droplet contacting the oil-wet glass surface.

In the first step, the micromodel was washed with sodium hydroxide solution and saturated for 30 min. Next, the vacuum pump evacuated the micromodel from any trapped fluid or air. To ensure that no fluid was trapped in the micromodel, the micromodel was washed with DW and dried in an oven at 200 °C. The micromodel was saturated with 2% hexamethyldisilane and 98% toluene for 30 min, as shown in Fig. 3, after which the glass surfaces became completely oil wet. Toluene and methanol were used to clean the micromodel. Finally, the micromodel was dried in an oven at 100 °C. All flooding tests were performed at atmospheric pressure and ambient temperature. The micromodel was placed horizontally to remove the effects of gravity^14^.

Flooding of the micromodel took place in three steps:

1. The micromodel was saturated with formation water.
2. The micromodel was saturated with oil.
3. The micromodel was flooded with injected fluids^29^.

**Figure 4:**
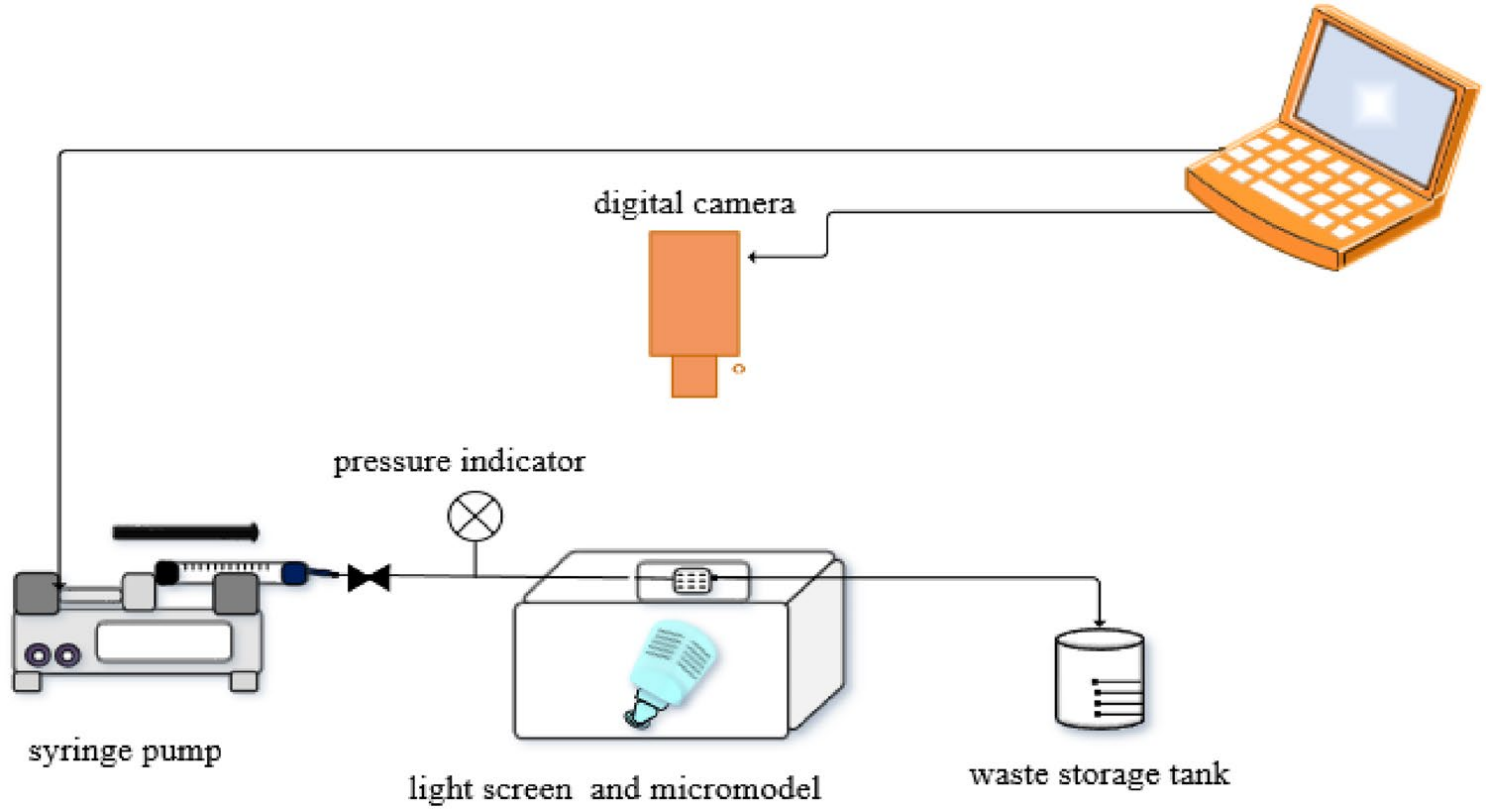
Schematic of the flooding setup.

All analyses performed on the micromodel were based on photographs taken. Micromodel photographs were taken every 0.2 PV for all the tests. Figure 4 shows that a syringe pump was used to injection the fluids. A digital microscope camera (digimicro1/3) that located above the micromodel was used for imaging. Finally, the images were analysed using image processing software to calculate the oil recovery factor. The oil recovery factor is calculated by Eq. 3, where the initial oil saturation (S_oi_) and the residual oil saturation (S_or_).

3 $$ Recovery = \left( {\frac{{\mathop S\nolimits_{oi} - \mathop S\nolimits_{or} }}{{\mathop S\nolimits_{oi} }}} \right) $$

## Results and discussion

### Stability evaluation of the nanosolutions

**Figure 5:**
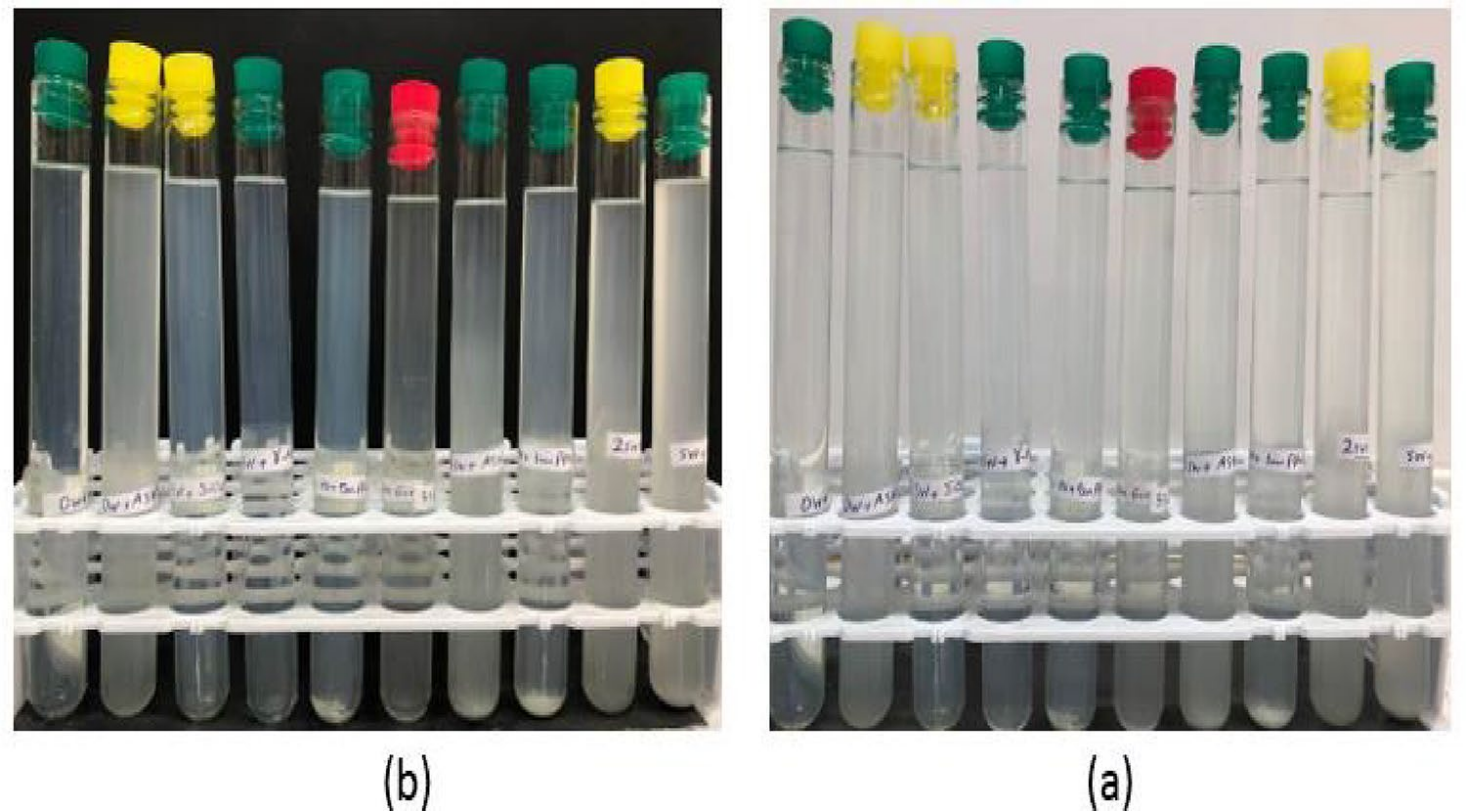
Photographs of NFs showing the stability for: (**a**) 2 h, (**b**) 2nd day.

Among the 25 NFs prepared, 15 had longer stability times. These nanosolutions with longer stability times were selected as the optimal NFs. In Table 5, the optimal NFs are shown with (*). The stability test was performed visually, similar to the methods of other researchers^21,28,48^. Sediment formation was observed at intervals of time on day one (1) and day sixty-third (63). In Fig. 5, photos of the stabilities of the different NFs are shown. (a) Two hours after preparation (b) 48 h after preparation. The NFs were considered stable when their dispersity remained constant in various base fluids, indicating that no sedimentation had formed. The interval time when sedimentation occurred at the end of the test tube and phase separation occurred at the top of the laboratory tube was considered the instability of the NFs.

**Table 5:** The results were obtained from the duration of stability.

Test no	Factor A: base fluid salt concentration (ppm)	Factor B: mass fraction γ-Al_2_O_3_ to SiO_2_ (AS)	Factor C: GA concentration (ppm)	Response: stability, (minutes)	Screening (selection of optimal NFs)
1	1	SiO_2_	0	89,280	*
2	1	AS_10:90_	250	3025	*
3	1	AS_30:70_	500	1540	*
4	1	AS_50:50_	750	1305	*
5	1	γ-Al_2_O_3_	1000	1002	*
6	4071	SiO_2_	250	40,574	*
7	4071	AS_10:90_	500	2130	*
8	4071	AS_30:70_	750	377	*
9	4071	AS_50:50_	1000	290	*
10	4071	γ-Al_2_O_3_	0	10	
11	8142	SiO_2_	500	21,267	*
12	8142	AS_10:90_	750	1235	*
13	8142	AS_30:70_	1000	270	*
14	8142	AS_50:50_	0	2	
15	8142	γ-Al_2_O_3_	250	141	
16	20,400	SiO_2_	750	4125	*
17	20,400	AS_10:90_	1000	485	*
18	20,400	AS_30:70_	0	17	
19	20,400	AS_50:50_	250	60	
20	20,400	γ-Al_2_O_3_	500	55	
21	40,710	SiO_2_	1000	3923	*
22	40,710	AS_10:90_	0	140	
23	40,710	AS_30:70_	250	117	
24	40,710	AS_50:50_	500	25	
25	40,710	γ-Al_2_O_3_	750	63

In rows 10, 14, 18, and 22 of Table 5, NFs for which GA was not used as a surfactant are shown. The duration of their stability was reported to be less than 2 h. However, the addition of different surfactants, such as ionic sodium dodecyl benzene sulfonate (SDBS) and CTAB, nonionic surfactants (Span 80, Tween 20) and polymers polyvinyl alcohol (PVA) and GA during NF preparation can also control particle aggregation^49^. This finding could indicate that the increase in stability with surfactant coating on NPs leads to the dominance of electrostatic repulsion over van der Waals attraction and thus prevents the accumulation of NPs.

According to the Derjaguin–Landau–Verwey–Overbeek (DLVO) theory, two forces cause the dispersion of particles: the van der Waals force of attraction and the electrostatic repulsion force of the double layer. The aggregation of NPs leads to their instability and in the case of nonaggregation their stability is maintained.

Salinity causes the instability of nanosuspensions^50^. In the rows 21–25 of Table 5, the NFs dispersed in SSW had the lowest stability. However, the NFs dispersed in DW had the highest stability compared to that of all the prepared nanosolutions. The first row of Table 5 shows that the SiO_2_ NPs were stable in DW without surfactant for 63 days. Additionally, according to the results of Zulkeflee and Mamat^51^ the addition of GA surfactant to silica NPs dispersed in DW improves the stability time compared to dodecylbenzene sulfonic acid (DBSA) and Chinese ink (CI) surfactants. In the 21st row of Table 5, silica NPs were dispersed in SSW with 1000 ppm GA and were stable for 65 h. According to the results of Hamdi et al.^52^ the functional groups of GA modify the surface of NPs and reduce the effect of van der Waals forces between nanosheets. As a result, more stable NFs were created even in high-salinity environments for a longer time. In row 5 of Table 5, the maximum stability time was 16 h for γ-Al_2_O_3_ NPs dispersed in DW with 1000 ppm GA.

Divalent cations such as Ca^2+^ can effectively neutralize the negative charge of SiO_2_ NPs. Therefore, when the van der Waals force of attraction between particles is greater than the electrostatic repulsion force, the accumulation and instability of the solution occur^53^. HNFs exhibit different behaviours when dispersed in DWs and brines. They are completely hydrophilic when dispersed in DW and have good stability, while they tend to cause sedimentation in brine. However, adding surfactants to HNFs results in a smaller aggregation size than in the absence of a surfactant^54^. The presence of monovalent cations such as Na^+^ in brine reduces the negative surface charge on silica NPs. Because silica NPs absorb monovalent cations such as Na^+^ present in brine, the stability of the solution decreases^17^. The dispersion of divalent ions such as Ca^2+^ and Mg^2+^ in brine results in amphoteric properties in HNFs^6^.

**Figure 6:**
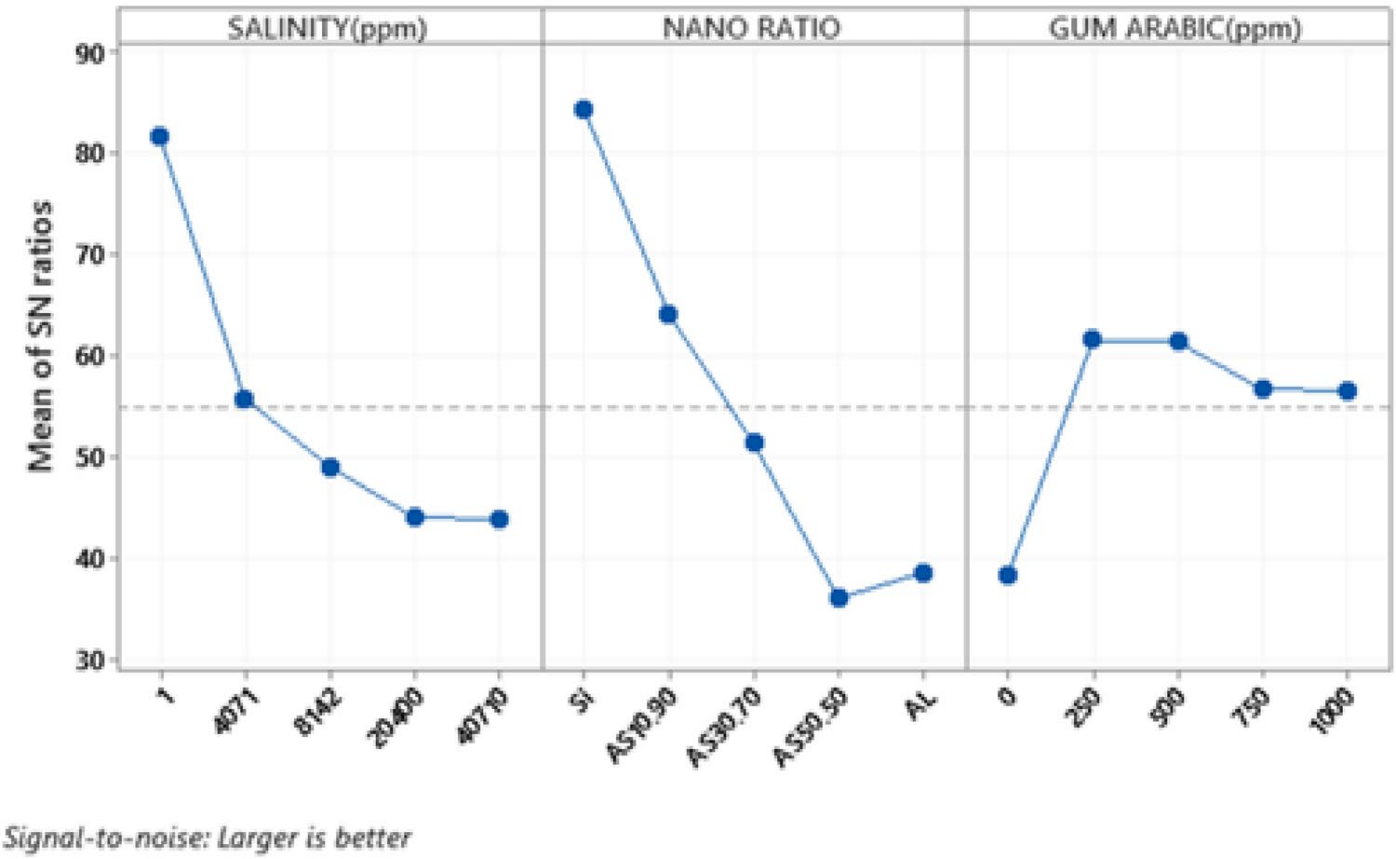
Results obtained from the taguchi method and the quality characteristic was selected large-the better. To investigation the salinity, the mass ratio of γ-Al_2_O_3_ to SiO_2_ and concentration of GA in NFs.

Three factors, the base-fluid salt concentration, mass fraction of nanosolutions and GA concentration, were studied to investigate the duration of stability, as shown in Fig. 6. The slope of the salinity graph could indicate an increase in the concentration of polyvalent ions. The results showed that the stability of HNFs with different mass fractions decreased with increasing amounts of alumina NPs, which could be due to the tendency of alumina to aggregate. Therefore, it is necessary to use stabilizers for the dispersion of alumina NPs^12^. Throughout the stability evaluation of the nanosolutions dispersed in the base fluid types, the highest stabilities were as follows: SiO_2_ NPs > combination of γ-Al_2_O_3_/SiO_2_ with a mass fraction of 10:90 > combination of γ-Al_2_O_3_/SiO_2_ with a mass fraction of 30:70 > γ-Al_2_O_3_ NPs > combination of γ-Al_2_O_3_/SiO_2_ with a mass fraction of 50:50. According to the results shown in Fig. 6, the most suitable concentration for GA was reported 250 ppm. At low surfactant concentrations, due to the Gibbs adsorption effect, surfactant molecules are absorbed at the fluid‒solid interface, which causes a decrease in the surface energy between the fluid and NPs, ultimately leading to a decrease in the aggregation of NPs^54^.

### IFT measurement

**Figure 7:**
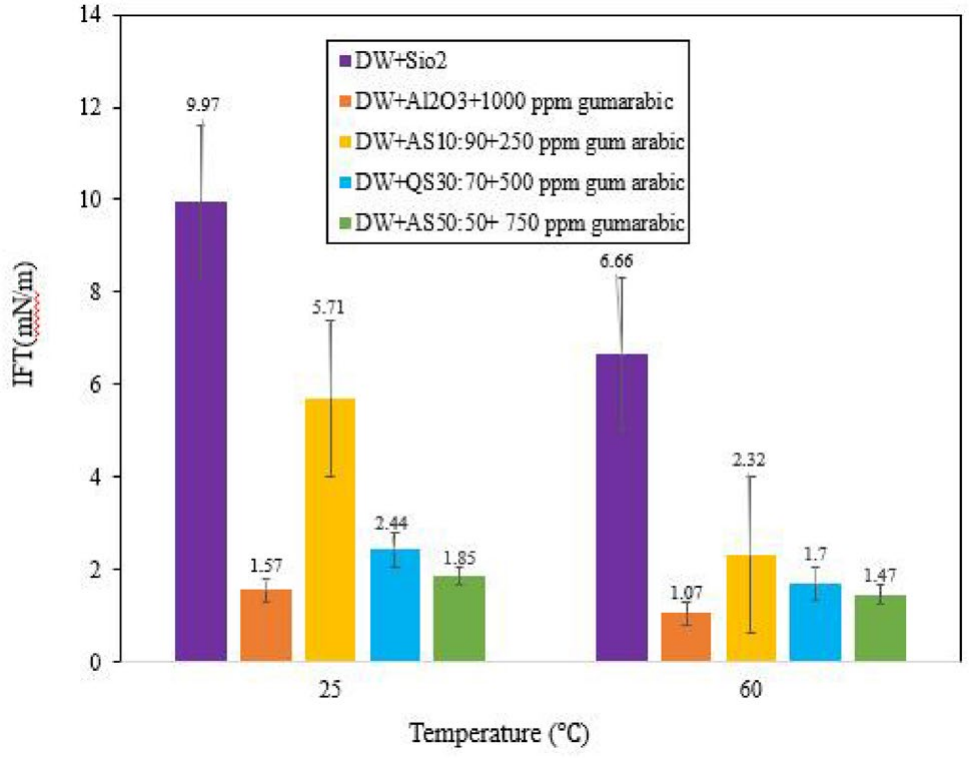
The effect of temperature on IFT of optimal NFs dispersed in DW. At ambient and 60 °C.

Any change in water composition (ionic or nonionic) could change the IFT. All IFT tests were performed at constant pressure. To ensure the reproducibility of the results and to determine the standard error, IFT measurements were performed at least seven times for the optimized NFs. According to the results of Afzalitabar et al.^55^ adding HNFs reduces the IFT. Figure 7 shows the IFT of NFs at 25 °C. HNFs γ-Al_2_O_3_/SiO_2_ with different mass fractions have lower IFT than SiO_2_ NPs; however, their IFT is greater than that of γ-Al_2_O_3_ NPs. Figure 7 shows that the IFT of NFs decreases as the temperature increases from 25 to 60 °C. At high temperatures, the IFT decreases, which could be due to an increase in the brownian motion of NPs or the weak interaction between molecules, ultimately leading to improved oil recovery^56^. Figure 7 shows that with increasing temperature from 25 to 60 °C, the IFT for SiO_2_ NPs decreased from 9.97 (mN/m) to 6.66 (mN/m) and that for γ-Al_2_O_3_ decreased to 1.57 (mN/m) and 1.07 (mN/m), respectively. In another attempt, Nowerouzi et al.^57^ reported that among the NPs of TiO_2_ and MgO and γ-Al_2_O_3_, the most successful at reducing the IFT was related to γ-Al_2_O_3_. Figure 7 shows the IFT with increasing temperature from 25 to 60 °C for HNFs with mass fractions of 10:90, 30:70 and 50:50 and GA concentrations of 250, 500 and 750 ppm, from 5.71, 2.44 and 1.85 (mN/m) to 2.32, 1.7 and 1.47 (mN/m), respectively. The presence of NPs modified with GA could reduce the IFT between crude oil and displacing nanofluid. Adsorption of modified NPs with GA at the interface between oil and brine occurs due to the hydrophilic and hydrophobic functional groups of the modified NPs, which cause the modified NPs to act as amphiphilic surfactants. Finally, the functional groups of the modified NPs create an additional layer at the interface between brine and oil to reduce the IFT^52^.

The effect of salt on IFT values in oil-salt water-containing systems is complex. A water-in-oil emulsion is formed in the presence of surfactants at low salt concentrations. In this system, increasing the amount of salt causes a further decrease in IFT. An oil-in-water emulsion is formed at high salt concentrations and the IFT increases with increasing salt concentration^58,59^. Adsorption of cations at the interface occurs due to the interaction between cations and the hydrocarbon phase. The interface concentration increases or becomes positive due to the placement of cations at the interface. As a result, the IFT decreases with decreasing salinity. However, at high brine concentrations, the cations are surrounded by water molecules, so the cations are less inclined to transfer to the interface. Separation of cations from the interface could reduce the positive charge of the interface. As a result, the IFT increases at high brine concentrations^59^.

**Figure 8:**
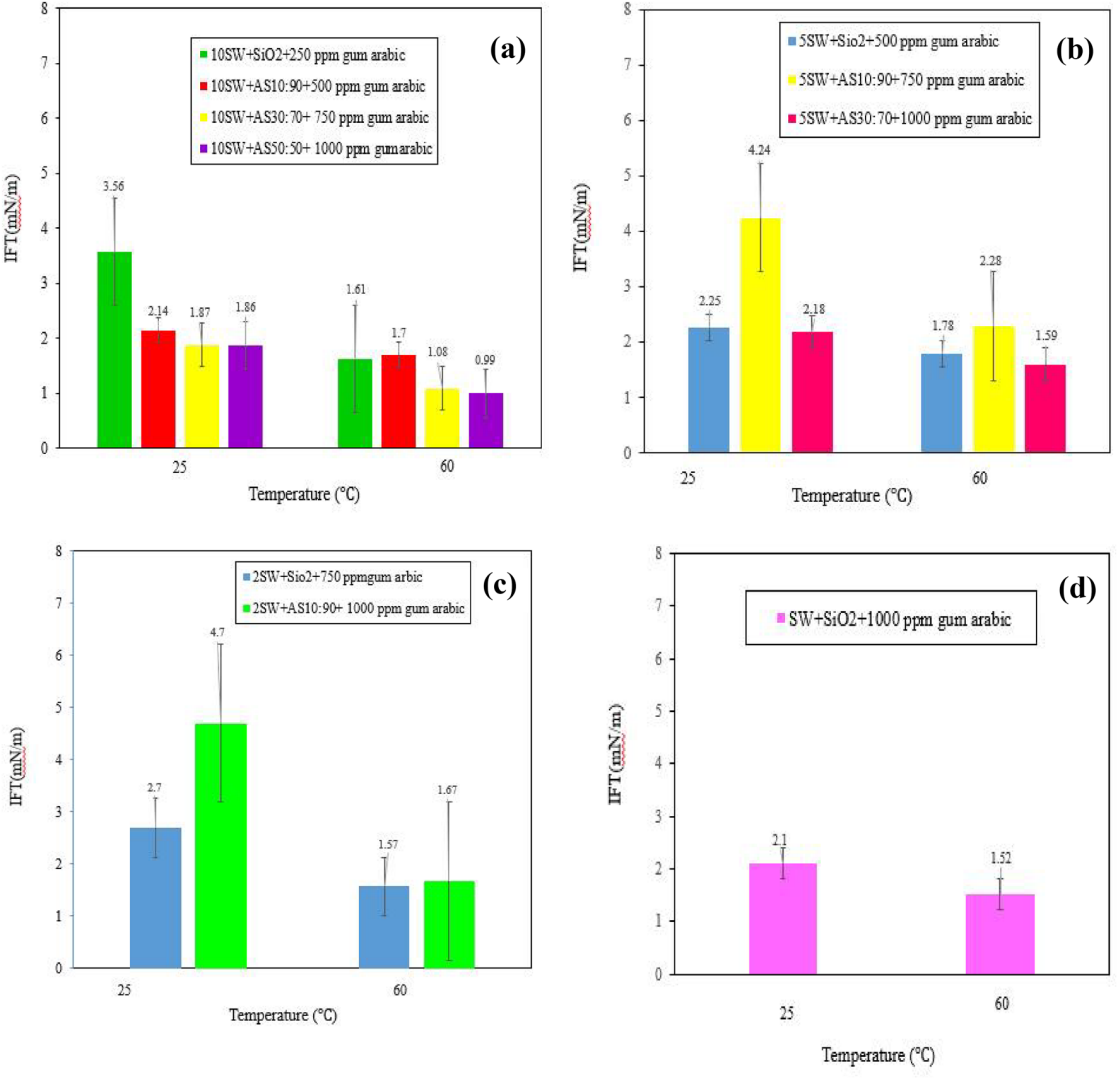
The effect of temperature on the IFT of optimal NFs dispersed in various salinities. At ambient and 60℃. (**a**) NFs dispersed in 10-DSSW. (**b**) NFs dispersed in 5-DSSW. (**c**) NFs dispersed in 2-DSSW. (**d**) NF dispersed in SSW.

Figure 8a shows the IFT of the NFs dispersed in 10-DSSW at ambient temperature and 60 °C. As shown, the NFs dispersed in 10-DSSW at 25 °C had a lower IFT than did the NFs dispersed in DW. This could be due to the reduction in IFT at low salt concentrations. The combination of HNF γ-Al_2_O_3_/SiO_2_ with a mass fraction of 50:50 modified with GA dispersed in 10-DSSW had the minimum IFT of 0.99 (mN/m) in 60 °C compared to that of the other NFs. However, Khaksar et al.^60^ reported the lowest IFT for silica-bentonite nanocomposite treated with anethum graveolens surfactant dispersed in 10,000 ppm NaCl at 1.73 (mN/m).

By increasing temperature, SiO_2_ NPs and HNFs with mass ratios of 10:90 and 30:70 were modified with GA (250, 500 and 750 ppm) respectively, their IFT decreased 1.57, 1.7 and 1.08 (mN/m). However, the results showed that the NFs dispersed in 10-DSSW had the lowest IFT.

Figure 8b shows the IFT of the NFs dispersed in 5-DSSW at ambient temperature and 60 °C. SiO_2_ NPs and HNFs with mass ratios of 10:90 and 30:70 were modified with GA (500, 750 and 1,000 ppm) and their IFT at 25 °C decreased from 2.25, 4.24 and 2.18 (mN/m) to 1.78, 2.28 and 1.59 (mN/m) respectively, at 60 °C.

Figure 8c shows that with increasing salinity of the base fluid, the IFT of the SiO_2_ NPs and HNF γ-Al_2_O_3_/SiO_2_ dispersed in 2-DSSW increased at ambient temperature compared to that of the NPs dispersed in 5-DSSW. This increase in the IFT of the NFs dispersed in 2-DSSW could be due to the high concentration of salt. As mentioned, increasing the salt concentration could reduce the separation of cations from the interface. Therefore, the silica NPs and HNF γ-Al_2_O_3_/SiO_2_ dispersed in 2-DSSW at ambient temperature had an IFT of 2.7 and 4.7 (mN/m) respectively. On the other hand, with increasing temperature, the IFT of the NFs dispersed in 2-DSSW decreased. However, in Fig. 8d, the IFT for SiO_2_ NPs dispersed in SSW modified with 1,000 ppm GA at ambient temperature and 60°C was reported 2.1 and 1.52 (mN/m), respectively.

### Viscosity measurement

**Figure 9:**
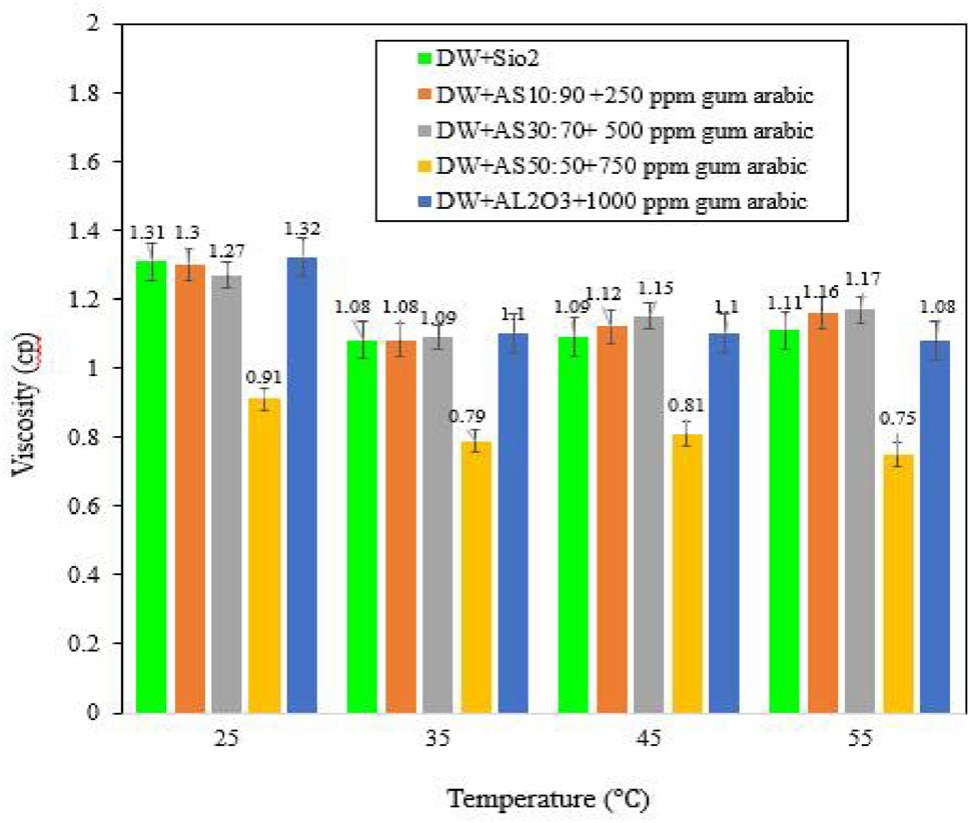
The effect of increasing temperature on the viscosity of NFs dispersed in DW.

The dynamic viscosities of the optimal nanosolutions were measured at temperatures of 25 °C, 35 °C, 45 °C and 55 °C. As shown in Fig. 9, the NFs dispersed in DW had lower viscosities than the NFs dispersed in various salinities. The results of Hashemzadeh and Hormozi^34^showed that γ-Al_2_O_3_ and SiO_2_ NPs dispersed in DW have relatively the same viscosity in the temperature range 10–55 °C. However, Fig. 9 shows that γ-Al_2_O_3_ NPs have a higher viscosity than SiO_2_ NPs. However, with increasing temperature, the viscosity of γ-Al_2_O_3_ decreases significantly compared to that of silica. The reason for this difference could be the presence of GA, which was applied together with the γ-Al_2_O_3_ NPs dispersed in DW at a mass ratio of 1:1. However, no surfactant was used with SiO_2_ NPs. As the temperature increases, the viscosity of the NFs decreases^61,62^. As inferred by Ma et al.^54^ adding surfactant improves nanofluid viscosity. According to the results of Buckley and Leverett^63^, decreasing mobility of the displacing fluid delays the breakthrough time. Additionally, the brownian motion of NPs induces interactions with surface-active compounds at the water–oil interface by attracting natural surfactants at the interface, thus reducing the IFT and improving oil recovery^14^. The reduction in nanocolloidal viscosity largely depends on the NPs and base fluids used. HNFs have a higher viscosity than do normal fluids and nanofluids. However, the viscosity of HNFs depends on the selected NPs and their composition^62^.

HNFs with mass ratios of 10:90 and 30:70 had lower viscosities than SiO_2_ and γ-Al_2_O_3_ NPs at low temperatures. However, with increasing temperature, the viscosity of these NFs increased compared to that of mono NFs. However, for HNFs a mass ratio of 50:50 was reported to result in a lower viscosity than that of mono nanofluids and this trend was observed at all the measured temperatures. In general, the viscosity of HNFs is greater than that of base fluids and single NPs^34,62^.

**Figure 10:**
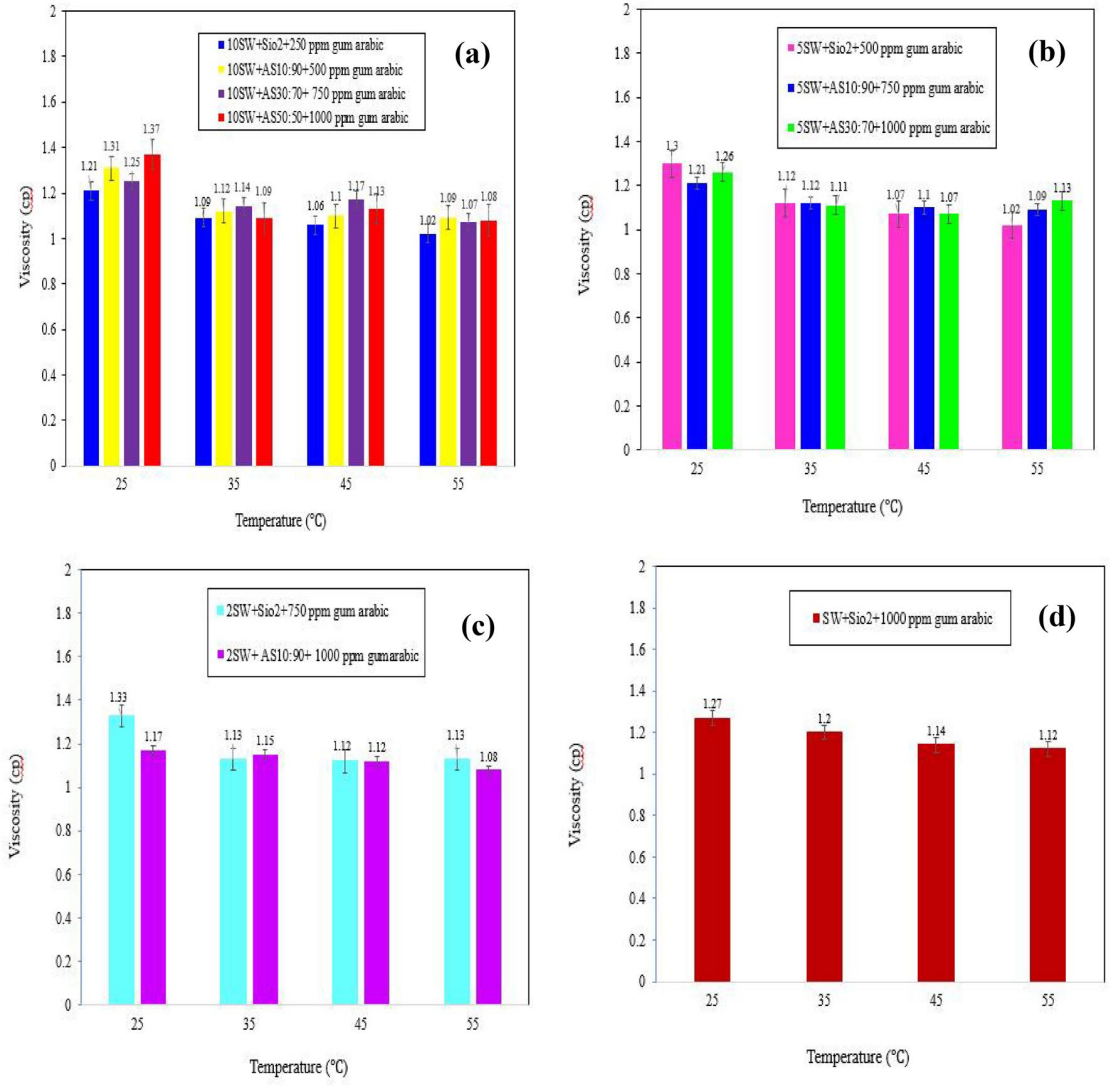
The effect of increasing temperature on the viscosity of NFs dispersed in various salinities. (**a**) NFs dispersed in 10-DSSW. (**b**) NFs dispersed in 5-DSSW. (**c**) NFs dispersed in 2-DSSW. (**d**) NF dispersed in SSW.

Figure 10a shows the viscosity as a function of temperature for SiO_2_ NPs and HNFs with mass ratios of 10:90, 30:70 and 50:50 modified with 250, 500, 750 and 1,000 ppm GA dispersed in 10-DSSW. The results show that HNFs with different mass ratios at different temperatures (25, 35, 45 and 55 °C) have higher viscosities than silica NPs and the lowest viscosity is obtained for SiO_2_ NPs at 55 °C. However, the combination of γ-Al_2_O_3_/SiO_2_ dispersed in 10-DSSW with a mass fraction of 50:50 has a higher viscosity than other nanosolutions at 25 °C. Although, by increasing temperature the combination of γ-Al_2_O_3_/SiO_2_ with a mass fraction of 50:50 the measured viscosity difference between 25 and 55 °C was reported 0.29 cp, which was observed greater than that of another HNFs with mass ratios of 10:90 and 30:70.

Figure 10b shows that the silica NPs dispersed in the 5-DSSW modified with 500 ppm GA at 25 °C had a greater viscosity than did the HNFs with mass ratios of 10:90 and 30:70 that, modified with 750 and 1000 ppm GA. However, with increasing temperature to 35 °C, the viscosities of the silica NPs and HNFs are somewhat equal. At high temperatures, the viscosity of HNFs was greater than the viscosity of silica NPs. The results showed that silica NPs and HNFs with a mass ratio of 10:90 exhibited a decreasing trend with increasing temperature, while the lowest viscosity was reported for the combination of HNF γ-Al_2_O_3_/SiO_2_ with a mass fraction of 30:70 at 45 °C.

Figure 10c shows that the HNF with a mass ratio of 10:90 and 1,000 ppm GA dispersed in 2-DSSW, compared to the viscosity of the SSW at 25 °C and 55 °C, has a lower viscosity than the silica NPs with 750 ppm GA. At 45 °C, the viscosities of the combinations of HNF γ-Al_2_O_3_/SiO_2_ with a mass fraction of 10:90 and silica NPs were equal. Moreover, the viscosity of the optimal NFs dispersed in 2-DSSW decreased. As shown in Fig. 10d, the viscosity of the silica NPs dispersed in SSW decreased with increasing temperature.

### Flooding tests

**Figure 11:**
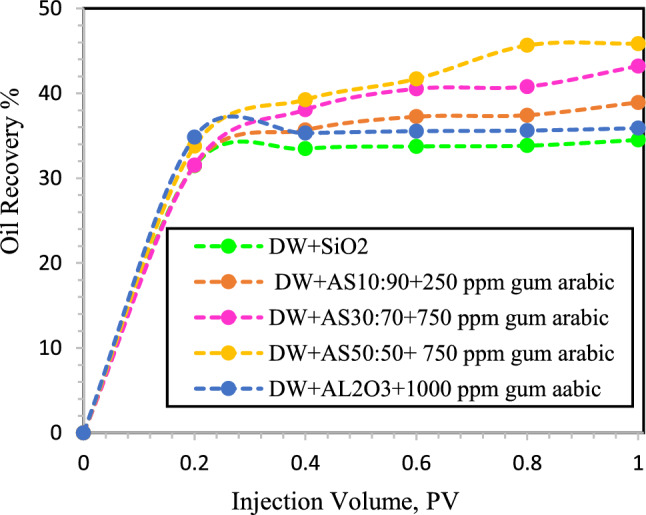
Oil recovery during injection of different NFs in to the oil-wet micromodel. In this section, SiO_2_, γ-Al_2_O_3_ NPs and HNFs γ-Al_2_O_3_/SiO_2_ with mass fractions (10:90, 30:70 and 50:50) modified with GA dispersed in DW.

Figure 11 shows that when NFs were dispersed in DW, breakthrough occurred after 0.4 PV. According to the results of the micromodel flooding, the minimum oil recovery was 34.4% when SiO_2_ NPs were injected into DW. Additionally, oil recovery was reported during the injection of γ-Al_2_O_3_ NPs (35.82%). According to the results of Nowrouzi et al.^57^ γ-Al_2_O_3_ NPs improve oil recovery by reducing the IFT and viscosity. However, during the injection of HNFs γ-Al_2_O_3_/SiO_2_ with mass fractions of 10:90, 30:70 and 50:50 and concentrations of 250, 500 and 750 ppm GA, oil recoveries of 38.92%, 43.2% and 45.85%, respectively, were obtained. In all the flooding tests, the synergistic effect of injecting HNFs was evident, and the oil recovery was greater than that of mono NFs. In similar studies, Khaksar et al.^60^ showed that SiO_2_/bentonite nanocomposites modified dill and hop surfactants can improve the oil recovery. The modified nanocomposite with 4000ppm dill extract can improve oil recovery by 14% of original oil in place. Their results confirmed the effectiveness of the formulated green nano-solution as a sustainable and consistent method with the environment in the EOR process.

HNFs increase oil recovery by transporting more residual oil. However, compared with water flooding, GA flooding improved the oil recovery factor on average by 4.6%. GA can reduce the IFT between brine and oil through its migration to the oil–water interface, where the hydrophobic polypeptide chain of GA reacts with oil and the hydrophilic arabinogalactan unit of GA reacts with water. Additionally, adding GA to silica and alumina NPs improved the oil recovery factor by 14.4% and 20.9%, respectively^64^.

**Figure 12:**
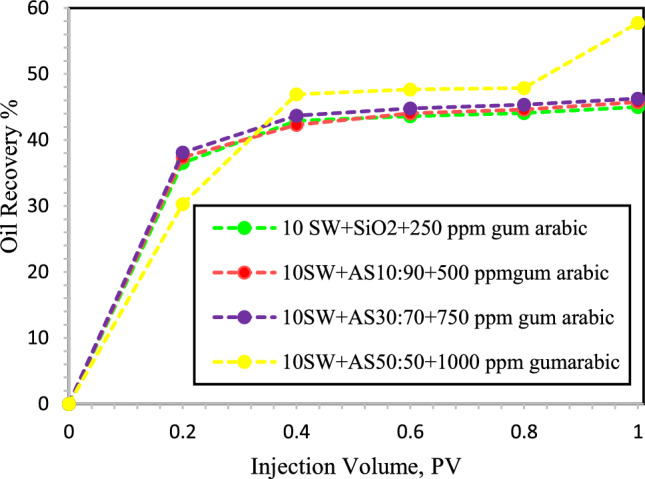
Oil recovery during injection of different NFs in to the oil-wet micromodel. In this section, SiO_2_ NPs and HNFs γ-Al_2_O_3_/SiO_2_ with mass fractions (10:90, 30:70 and 50:50) modified with GA dispersed in 10-DSSW.

Figure 12 shows that breakthrough occurs at 0.5 PV when the NFs are dispersed in 10-DSSW. The oil recoveries during the injection of SiO_2_ NPs and HNFs with mass fractions of 10:90, 30:70 and 50:50 along with different concentrations of GA were reported as 45, 45.75, 46.28 and 57.75%, respectively. We observed that, among the optimized NFs that were stably dispersed in 10-DSSW, HNF γ-Al_2_O_3_/SiO_2,_ with a mass fraction of 50:50 and modified with 1,000 ppm GA, initially had lower oil recovery than the other NFs. However, after 0.8 PV, the oil recovery of the HNF γ-Al_2_O_3_/SiO_2_ composite increased significantly.

**Figure 13:**
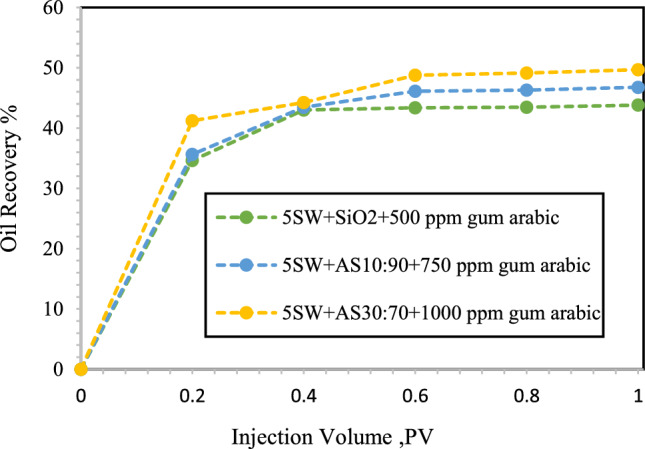
Oil recovery during injection of different NFs in to the oil-wet micromodel. In this section, SiO_2_ NPs and HNFs γ-Al_2_O_3_/SiO_2_ with mass fractions (10:90 and 30:70) modified with GA dispersed in 5-DSSW.

As evident in Fig. 13, a delayed breakthrough occurred when the NFs dispersed in 5-DSSW were injected in comparison to the NFs dispersed in 10-DSSW, and the breakthrough time occurred at 0.6 PV. The oil recovery during the injection of SiO_2_ NPs and HNFs with mass fraction 10:90 and 30:70 and modified with 500, 750 and 1000 ppm GA were reported 43.8, 46.77 and 49.68%, respectively. We observed in micromodel flooding tests that increasing the concentration of brine can improve oil recovery. According to the results of Rostami et al.^4^, NPs dispersed in brines have a better performance in improving the oil recovery compared to NPs dispersed in deionized water. NPs flooding modified with GA compared with water flooding, has a greater ability to sweep out oil. The ability of the modified NPs to reduce the IFT between the crude oil and nanofluid solutions ultimately reduced the capillary pressure inside the pores. Additionally, the modified nanofluids alter the wettability of the pores surface to strong water wetting^52^.

**Figure 14:**
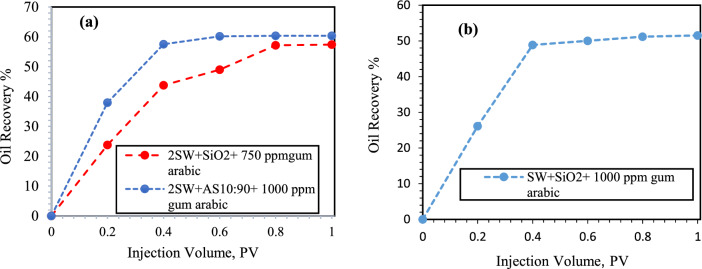
Oil recovery during injection of different NFs in to the oil-wet micromodel. (**a**) SiO_2_ NPs and HNFs γ-Al_2_O_3_/SiO_2_ with mass fractions 10:90 modified with GA dispersed in 2-DSSW. (**b**) SiO_2_ NPs modified with GA dispersed in SSW.

Figure 14a shows that, for NFs dispersed in 2-DSSW breakthrough occurs delayed at 0.8 PV compared to NFs dispersed in 5-DSSW. We observed a maximum oil recovery of 60.34% when the combination of γ-Al_2_O_3_/SiO_2_ was injected with a mass fraction of 10:90 dispersed in 2-DSSW. On the other hand, Khaksar et al. were achieved the maximum oil recovery factor for KCl–SiO_2_–xanthan nanocomposite treated with the aloe vera biopolymer surfactant at 73.35%. They also attributed the increase in oil recovery to the reduction of IFT, wettability alteration and mobility improvement mechanisms^65^. Figure 14b shows that by injecting 0.1 wt% SiO_2_ nanofluid dispersed in SSW into the micromodel, 51.53% oil recovery was obtained.

**Figure 15:**
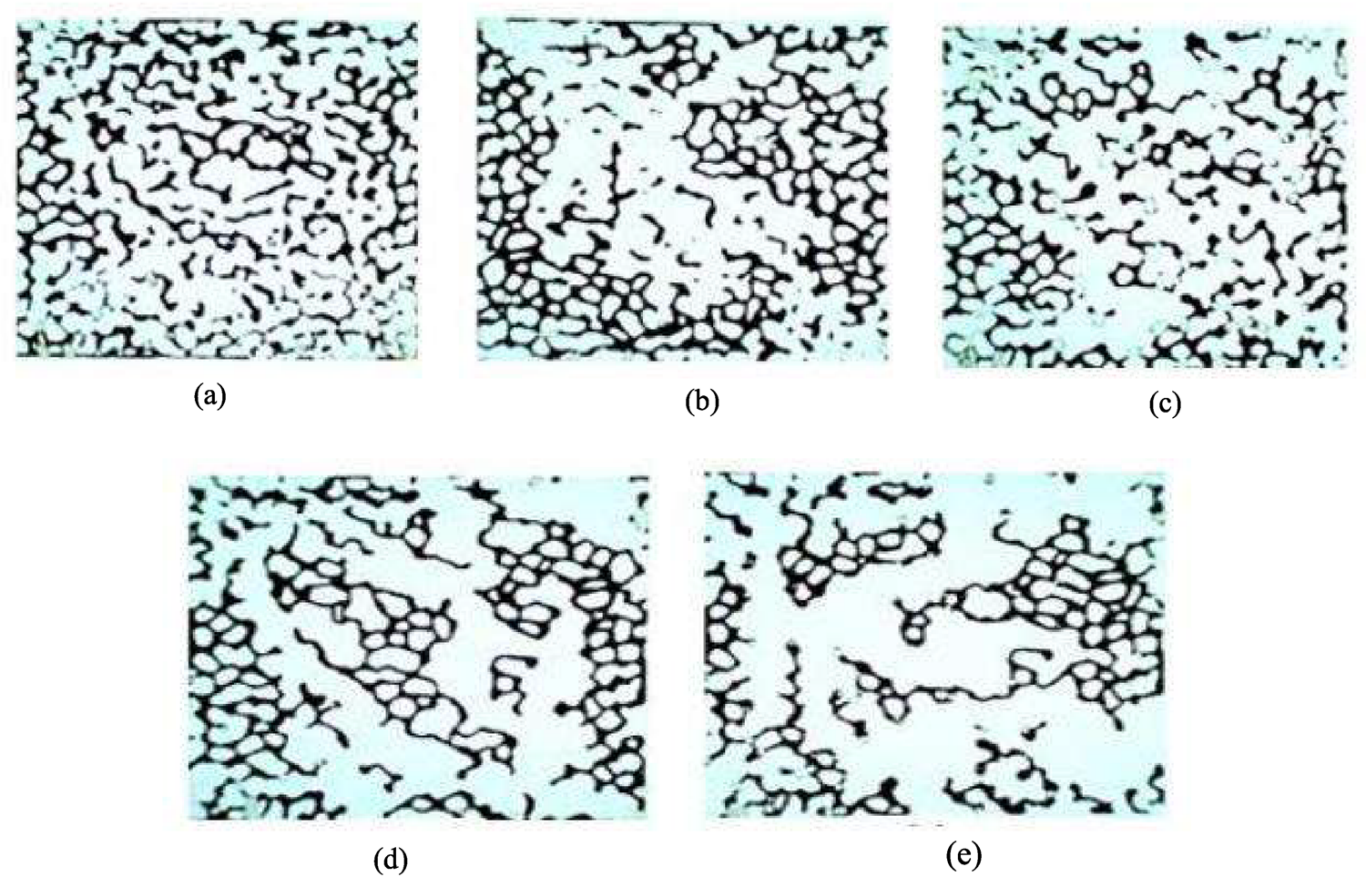
Oil recovery for different NFs after 1 PV the injection. (**a**) SiO_2_ NPs dispersed in DW without GA. (**b**) γ-Al_2_O_3_ NPs dispersed in DW modified with GA. (**c**) HNF γ-Al_2_O_3_/SiO_2_ with a mass fraction of 30:70 dispersed in 5-DSSW modified with GA. (**d**) HNF γ-Al_2_O_3_/SiO_2_ with a mass fraction of 50:50 dispersed in 10-DSSW modified with GA. (**e**) HNF γ-Al_2_O_3_/SiO_2_ with a mass fraction of 10:90 dispersed in 2-DSSW modified with GA.

Figure 15 shows micromodel images used for sweep efficiency calculations. The images show the injection of some NFs after the injection of 1 PV into the treated to oil-wet micromodel. From these images, it could be concluded that the injection of HNF γ-Al_2_O_3_/SiO_2_ in combination with a mass fraction of 10:90 dispersed in 2-DSSW with 1,000 ppm GA into the micromodel had the highest areal sweep efficiency. Figure 15e shows the synergistic effect of HMF γ-Al_2_O_3_/SiO_2_ modified with GA dispersed in 2-DSSW, which increased the displacement efficiency of the oil mixture among the other injection scenarios. Figure 15a also shows that the silica NPs dispersed in DW without GA had the lowest oil recovery among the other optimal NFs.

## Conclusions

In this experimental study, the effects of mono/hybrid γ-Al_2_O_3_/SiO_2_ nanofluids dispersed at various salinities and modified with GA surfactant on the stability, viscosity, IFT and oil recovery were investigated. The results showed that the NFs became highly unstable with increasing salt concentration to 40,710 ppm and quickly accumulated at the end of the test tube. However, the NFs that were not modified with GA became unstable after approximately 2 h. The stability of NPs in base fluid is essential for the long-term utilization of NFs, particularly for EOR applications.

In addition, the effects of temperature, various salinities of base fluid and mass fractions of γ-Al_2_O_3_/SiO_2_ on the viscosity and IFT of long-term stable NFs were evaluated. The results of the IFT evaluation showed that increasing temperature had a greater effect on the dispersal of NFs at various salinities than on the dispersion of NFs in DW. However, the results clearly showed that with increasing salinity to 20,400 ppm the IFT increased.

Also, the evaluation of viscosity results shows that with increasing temperature, NFs dispersed in DW have lower viscosity than NFs dispersed in various salinities. According to results of experimental viscosity data nanohybrids with different mass fractions by increasing temperature show different action in the presence of cations in salt water and deionized water. The lowest viscosity was observed at 55 °C for the nanohybrid γ-Al_2_O_3_/SiO_2_ with a mass fraction of 50:50 dispersed in deionized water.

The results of the micromodel flooding test showed that increasing the salinity of the base fluid leads to a delay in breakthrough. In this way, NFs dispersed in DW were observed at 0.4 PV breakthrough time; however, NFs dispersed in 2-DSSW were reported at 0.8 PV breakthrough time.

## Data Availability

The datasets used and/or analysed during the current study available from the corresponding author on reasonable request.
